# Long-term effectiveness of elderly health care voucher scheme strategies: a system dynamics simulation analysis

**DOI:** 10.1186/s12889-021-11280-z

**Published:** 2021-06-26

**Authors:** Ka Chun Chong, Hong Fung, Carrie Ho Kwan Yam, Patsy Yuen Kwan Chau, Tsz Yu Chow, Benny Chung Ying Zee, Eliza Lai Yi Wong, Maggie Haitian Wang, Eng Kiong Yeoh

**Affiliations:** 1 The Jockey Club School of Public Health and Primary Care, Faculty of Medicine, The Chinese University of Hong Kong, Hong Kong, Special Administrative Region China; 2 Centre for Health System and Policy Research, Faculty of Medicine, The Chinese University of Hong Kong, Hong Kong, Special Administrative Region China

**Keywords:** Elderly healthcare voucher, Financial incentive, Chronic, Elderly care, Simulation, System dynamics

## Abstract

**Background:**

The elderly healthcare voucher (EHCV) scheme is expected to lead to an increase in the number of elderly people selecting private primary healthcare services and reduce reliance on the public sector in Hong Kong. However, studies thus far have reported that this scheme has not received satisfactory responses. In this study, we examined changes in the ratio of visits between public and private doctors in primary care (to measure reliance on the public sector) for different strategic scenarios in the EHCV scheme.

**Methods:**

Based on comments from an expert panel, a system dynamics model was formulated to simulate the impact of various enhanced strategies in the scheme: increasing voucher amounts, lowering the age eligibility, and designating vouchers for chronic conditions follow-up. Data and statistics for the model calibration were collected from various sources.

**Results:**

The simulation results show that the current EHCV scheme is unable to reduce the utilization of public healthcare services, as well as the ratio of visits between public and private primary care among the local aging population. When comparing three different tested scenarios, even if the increase in the annual voucher amount could be maintained at the current pace or the age eligibility can be lowered to include those aged 60 years, the impact on shifts from public-to-private utilization were insignificant. The public-to-private ratio could only be marginally reduced from 0.74 to 0.64 in the first several years. Nevertheless, introducing a chronic disease-oriented voucher could result in a significant drop of 0.50 in the public-to-private ratio during the early implementation phase. However, the effect could not be maintained for an extended period.

**Conclusions:**

Our findings will assist officials in improving the design of the EHCV scheme, within the wider context of promoting primary care among the elderly. We suggest that an additional chronic disease-oriented voucher can serve as an alternative strategy. The scheme must be redesigned to address more specific objectives or provide a separate voucher that promotes under-utilized healthcare services (e.g., preventive care), instead of services designed for unspecified reasons, which may lead to concerns regarding exploitation.

**Supplementary Information:**

The online version contains supplementary material available at 10.1186/s12889-021-11280-z.

## Background

Projection statistics from the Hong Kong Census Department [1] show that the population of Hong Kong is expected to increase to 8.22 million in 2043. Compared with 2011, the proportion of the population aged 65 years and older is expected to increase by 33% by 2064. With the increase of the aging population in Hong Kong, the overall public health expenditure is projected to be $127 billion by 2025 [2]. Chronic diseases, such as stroke, dementia, and diabetes, together with cognitive and physical decline associated with dying, will become increasingly prevalent due to this demographic change. The primary care system is becoming more important for managing patients with chronic diseases who require continuous care and treatment.

In Hong Kong, primary care is primarily fulfilled by the private sector. Among the elderly population aged 60 years or older, the proportion of those who consulted public primary care doctors was higher, compared with that of those who consulted private practitioners [3, 4]. Moreover, among patients with chronic conditions, the proportion of those utilizing public healthcare services is significantly higher. To develop a long-term strategy for enhancing the provision of elderly primary care services, the elderly healthcare voucher (EHCV) scheme was implemented in 2009 for a pilot period of 3 years. By offering financial incentives, the scheme allows elderly people to select private healthcare services as an alternative for relieving the burden on public healthcare services, such as general and specialist outpatient clinics. At the start of the EHCV scheme in 2009, five $50 (in HKD) vouchers were provided on an annual basis to each elderly person aged 70 years or older. After conducting a review of the scheme, the annual amounts were increased to $500 and $1000 in 2012 and 2013, respectively. In 2014, the amount was increased to $2000, and its cumulative amount was allowed to be carried forward up to $4000 for subsequent years [5]. In 2017, the age eligibility was lowered to include those aged 65 years or older.

Financial incentives, such as voucher programs, are common determinants of people’s choices regarding the use of public and private healthcare services [6–9]. The intervention helps to balance the demand for public and private services in a public–private mixed system [10, 11]. Theoretical studies have indicated that subsidies for private services can effectively reduce the demand for free public services in some circumstances [12]. Vouchers are a demand-side subsidy aimed to reduce financial problems faced when accessing services [13]. This is a useful method of targeting specific populations and can improve the quality of services by incentivizing behavior change on both the demand and supply sides [10, 14, 15]. Studies have shown evidence regarding the effectiveness of vouchers in encouraging people to perform clearly defined, time-limited, and simple behavioral tasks [10, 16, 17]. For example, the adolescent voucher program in Nicaragua led to increased accessibility of contraceptives through health services [16]. Vouchers for free mammography can significantly improve compliance rates in rural regions [17]. Some evaluations of voucher programs have reported positive associations with increased utilization [10, 18, 19]. Widespread acceptance of the governmental tool led the World Bank to issue a guide for vouchers; this guide identified the advantages of vouchers and highlighted the choices and decisions involved in using vouchers [13].

However, the effectiveness of the EHCV scheme remains a controversial topic. Yam et al. found that voucher usage was low during the initial phase of the EHCV scheme [20, 21]; only 35% of the elderly had used it primarily due to the low subsidy provided at that time. In particular, only approximately 7% of the health care vouchers claimed were from preventive services. In addition, the choices of healthcare providers were also limited, because the electronic platform for claiming was considered inconvenient by providers. According to an interim report from the Department of Health [22], the estimated participation rate of medical practitioners was only approximately 34.1% in 2011. Although enhancement has been adopted since the interim review, a survey conducted in 2015 revealed several problems, such as the low enrollment rate of private doctors [23]. Most respondents continued to believe that the number of vouchers should be increased. A retrospective cohort study indicated that voucher usage was not associated with reduced utilization of public healthcare services in the 2009–2015 period [24]. As with suggestions from the Nicaragua study [16], there is room for improvement in the voucher program—for example, sustaining a longer intervention period and enhancing quality improvements.

According to the Hong Kong government, the EHCV scheme is expected to increase the number of elderly people opting for private primary healthcare services to reduce reliance on the public sector. Studies have yet to sufficiently address the scheme [20–23, 25]. The current problem of the scheme is the difficulty related to affecting behavioral changes in a proportion of users and healthcare services in the short period of the pilot period. Therefore, a retrospective analysis may be unable to draw any significant impact on changes in the utilization of public healthcare services. The Hong Kong government continues to monitor the voucher scheme and refines it frequently to ensure that the goal of the scheme can be fulfilled. Therefore, a system dynamics (SD) modeling design can test the “what-if” scenarios when the EHCV scheme is subject to regular refinement in an aging population. Our study aims to evaluate the long-term effectiveness of the enhanced strategies of the EHCV scheme in reducing reliance on public primary care services.

## Method

### Objectives

The current study adopts a system dynamics model based on the settings of the EHCV scheme in Hong Kong. The objective of this modeling study is to examine changes in the ratio of visits between public and private doctors in primary care (as a metric of reliance on the public sector) based on different strategic scenarios.

### Design of scenarios

A qualitative phase was initiated to develop a conceptual model (i.e., stock-and-flow) as a preliminary sketch of the behavior system that illustrates the pathway of individuals using the voucher and factors affecting the loop, and to design scenarios for testing. The factors were identified based on the results of prior studies, the interim report of the EHCV scheme, a recent survey from the Hong Kong Medical Association, and some unpublished cross-sectional studies [20–23, 25]. An expert panel was formed to comment on the importance of the factors in the system of using vouchers and identify the adjustments or interventions that should be introduced. Five independent private primary care professionals who had worked in the private sector for a minimum of 10 years were invited to participate in interviews through the networks of the researchers. These professionals were also enrolled in the EHCV scheme and used the claiming system. Their comments were noted by Chong et al. [26] and scenarios for increasing voucher amounts, lowering the age eligibility criteria, and designating vouchers for chronic conditions follow-up were considered in the simulation testing.

The study ethics, as well as the verbal consent procedure, was approved by the Survey and Behavioral Research Ethics Committee of the Chinese University of Hong Kong. Verbal consent was also obtained from the participants.

### System dynamics model

An SD model was developed for computational simulations aimed at testing different strategic scenarios. In public health studies, SD modeling, involving the development of computational simulation, can address the dynamic complexity of different types of care in a health system and systematically evaluate policies for influencing changes. In our study, the SD model was stratified into five levels.

**Fig. 1:**
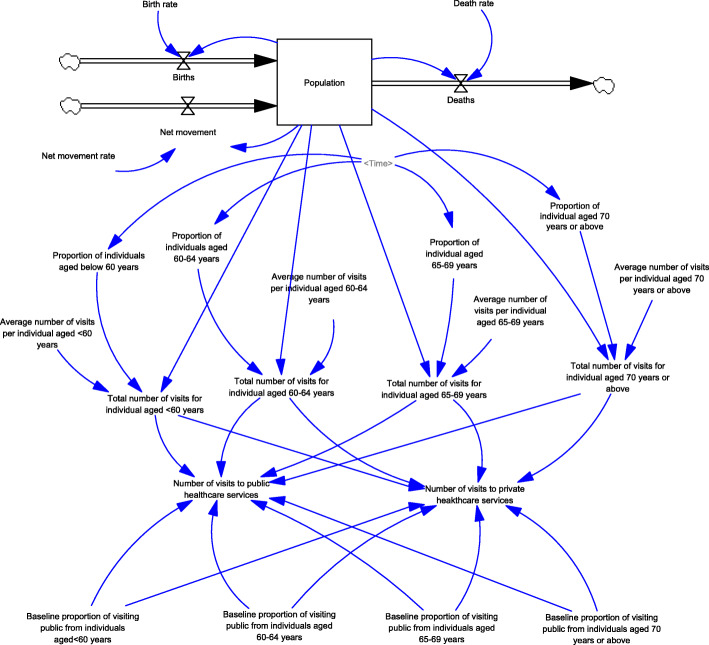
Diagram of the system dynamics model for generation of visits to healthcare services in a population

**Fig. 2:**
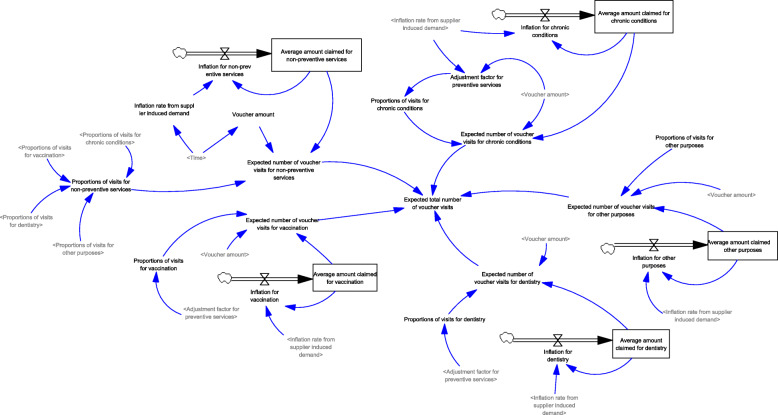
Diagram of the system dynamics model for generation of visits using EHCV

**Fig. 3:**
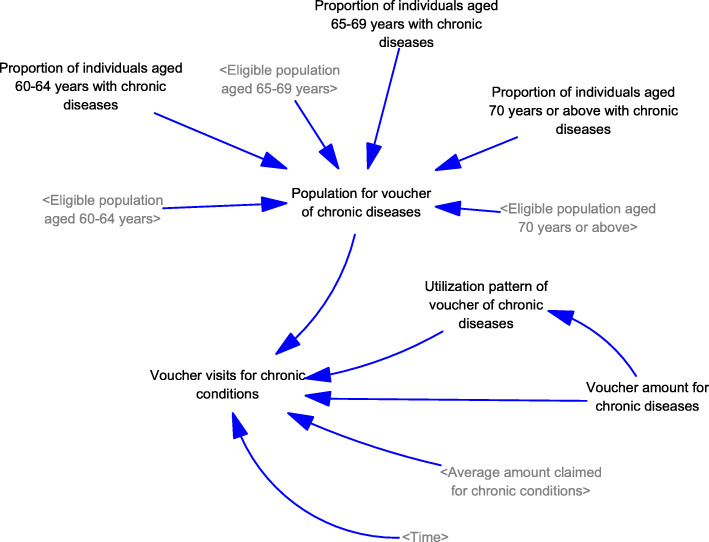
Diagram of the system dynamics model for generation of visits using vouchers for chronic diseases

**Fig. 4:**
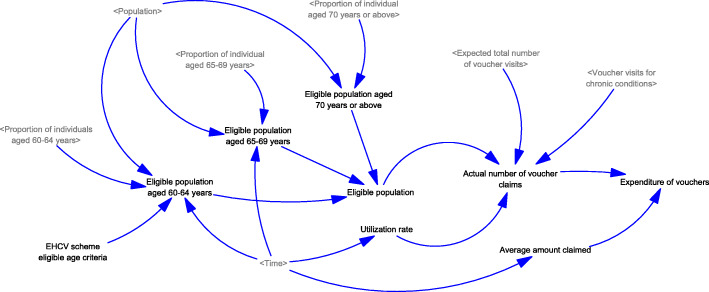
Diagram of the system dynamics model for generation of the actual number of visits using vouchers

**Fig. 5:**
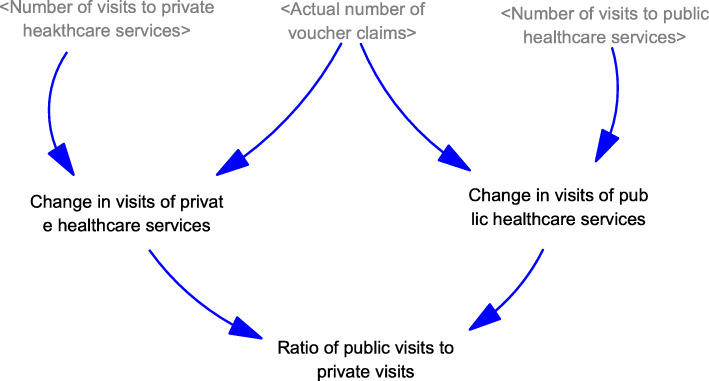
Diagram of the system dynamics model for changes in utilization of public healthcare services

- Generation of visits to healthcare services (Fig. 1): A population model, including new births and deaths, was developed. In the population model, the total number of visits from individuals of different age groups (i.e., < 60, 60–64, 65–69, and ≥ 70 years) were generated based on the average number of visits and population sizes. The number of visits to public and private healthcare services was generated assuming the baseline proportions of visits to public and private primary care services, which is independent of the use of vouchers.
- Generation of visits for using EHCV (Fig. 2): Based on the settings of the voucher (e.g., voucher amounts and the purposes of use), the expected number of visits for different services (i.e., non-preventive services, chronic conditions follow-up, dentistry, vaccination, and others) were generated. Inflation in service prices from supplier-induced demand was considered according to the trends reported in the historical claims information. The expected total number of voucher visits was the sum of the expected number of visits for different services.
- Generation of visits for using vouchers for chronic diseases (Fig. 3): A designating voucher for chronic condition follow-up was suggested for scenario testing. In the SD model, the total number of voucher visits for chronic conditions was determined based on the eligible population size with chronic diseases in different age groups and their utilization patterns. The utilization patterns of the voucher varied according to the voucher amount, based on the assumption collected from previous cross-sectional surveys.
- Generation of the actual number of visits using vouchers (Fig. 4): Based on the proportion of the eligible population willing to join the scheme, the actual number of visits was determined with the corresponding total expenditure for the model calibration.
- Changes in the utilization of primary healthcare services (Fig. 5): Based on utilization from the voucher uses, the net changes in visits to public and private primary care providers were calculated based on differences in the number of visits shifted from public to private primary care and the baseline number of visits in each sector. The ratio of visits between the public and private sectors was determined over time.

A set of corresponding deterministic and differential equations of the SD model was developed and computerized to present the flow of individuals seeking healthcare services in the public and private sectors. The unit of time in the simulation settings was set to weeks. Time-based inputs were repeated every week over a 15-year trend of aging (2017–2032). Euler’s method was used to solve differential equations. The utilization rate is the most widely used outcome measurement for different voucher programs [27]; therefore, the primary endpoint in the simulations is the ratio of visits to public and private doctors by year as a measure of reliance.

### Scenario settings

The settings of the tested scenarios were presumed to be as follows:

1. Increasing voucher amounts: We tested the annual voucher amounts increasing from $3000, to $4000, and $5000 in the years 2021, 2025, and 2029, respectively, thereby maintaining the rate of increment similar to the historical trends of the current EHCV scheme.
2. Lowering the age eligibility: We tested the inclusion of elderly people aged 60–64 years in the scheme, starting from the year 2021.
3. Designating vouchers for chronic conditions follow-up: In addition to the original EHCV, we tested an additional voucher for visits aimed at treating chronic conditions. An extra amount of $2000 was provided to the eligible population every year, starting from 2021, to treat chronic diseases.

### Data collection for model inputs

Data and statistics for the inputs of the SD model were collected from different sources.

- Demographics statistics were derived from the Hong Kong Census and Statistics Department [1, 28].
- Utilization statistics for public healthcare services were derived from the Hospital Authority Statistical Reports and Thematic Household Survey Report [3, 29, 30].
- Baseline EHCV statistics were derived from the literature, the interim report of the Department of Health (DH), the published survey from the Hong Kong Medical Association [18–22], and cross-sectional studies (unpublished). The cross-sectional studies included a repeated cross-sectional survey of elderly persons aged 70 or above assessing their changes in attitudes toward, and usage of, vouchers among elderly persons in the community. These studies also include a public opinion survey of the general public examining the potential use of vouchers in primary care systems (e.g., enhancement of vouchers for preventive care and chronic disease management from the general public perspectives).
- Statistics of claimed amounts were derived from the DH administrative data and other relevant published statistics [31, 32].

### Baseline calibration

The baseline scenario followed the current EHCV scheme: $250 annually for elderly people aged 70 years or above in 2009–2011, $500 in 2012, $1000 in 2013, $2000 in 2014–2016 annually, and $2000 annually for elderly people aged 65 years or above after 2016. For the calibration of the SD model, the model simulated total expenditure from voucher visits validated against the total expenditure on voucher claims from 2009 to 2016, as published by the DH [31, 32]. The mean absolute percentage error (MAPE) of the calibrated model should be maintained below 40%. The R-squared value was also obtained to assess the fitness of the model calibration. Based on the calibrated model, the numbers of visits that used vouchers for different services were simulated until 2032. The variable specifications for the model development are listed in Additional File 1.

## Results

### Baseline projections

**Fig. 6:**
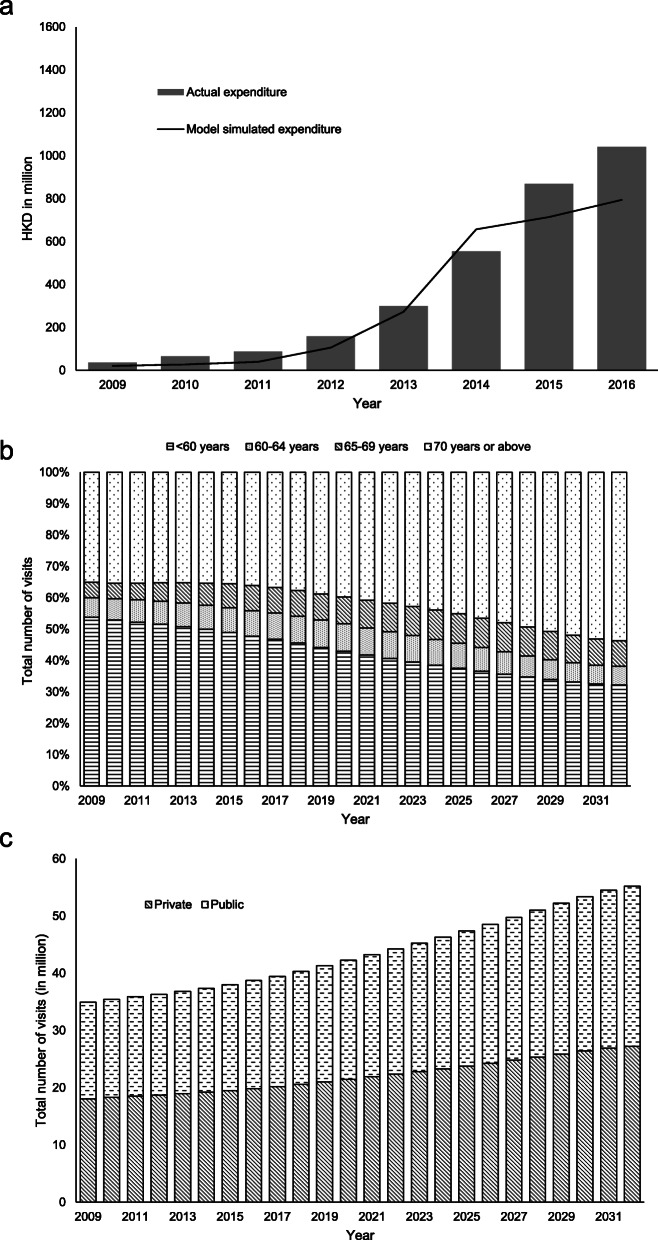
**a** Model-simulated expenditure and actual expenditure of voucher uses, **b** percentage of total visits by age group in the baseline scenario, and **c** comparison of total visits between public and private in the baseline scenario

Figure 6a presents the model-simulated expenditure and actual expenditure of voucher uses provided by DH. The model calibration showed acceptable fitness with MAPE = 30.5% and R-square = 92.1%.

In the population model, the total number of visits from individuals aged 70 years or above increased from 35% in 2009 to 54% in 2032, whereas the total number of visits from individuals aged below 60 years decreased from 54% in 2009 to 32% in 2032 (Fig. 6b). Due to the aging population, utilization of public healthcare services increased from 16.9 million in 2019 to 28.0 million in 2032, whereas the utilization of private healthcare services increased from 18.0 million in 2019 to 27.2 million in 2032 (Fig. 6c).

### Comparison of different tested scenarios

**Fig. 7:**
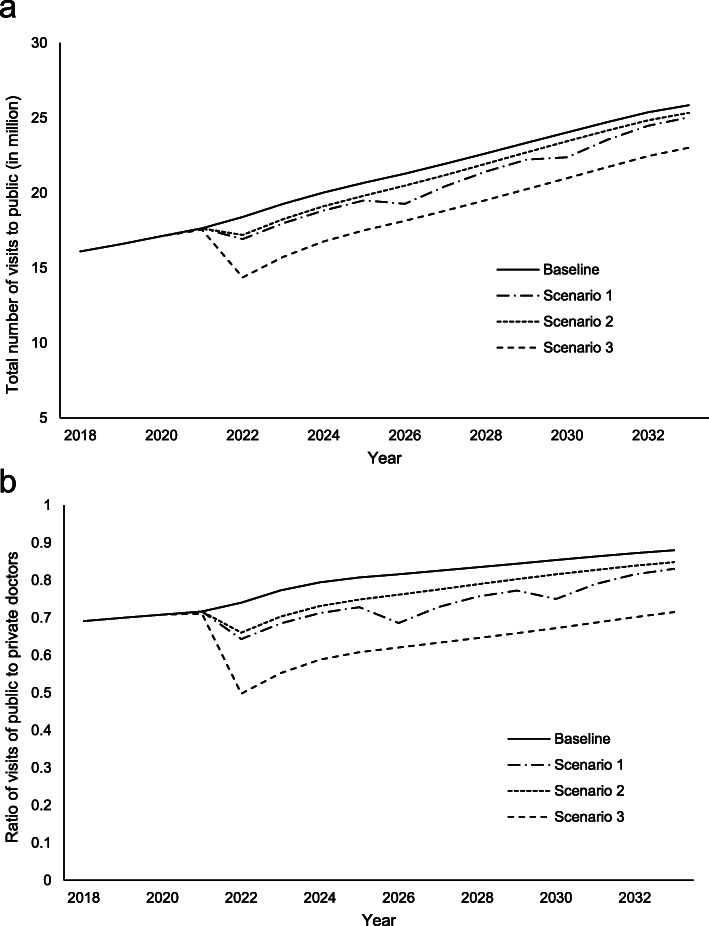
Changes in **a** public healthcare utilizations and **b** ratio of visits between public and private by years in different scenarios

Three scenarios of increasing voucher amounts, lowering the age eligibility, and designating vouchers for chronic conditions were tested in the SD model with plausible price settings of voucher amounts. The simulation results showed that the current EHCV scheme (i.e., $2000 annually for individuals aged 65 years or above, starting from 2017) was unable to reduce the utilization of public healthcare services and the ratio of visits between public and private primary care services when population aging was taken into account in the generation of visits to healthcare services (Fig. 7a and b). The total number of visits to public doctors increased from 16.3 million in 2018 to 25.6 million in 2032, whereas the public-to-private ratio of visits increased from 0.70 in 2018 to 0.88 in 2032.

Among the tested strategies (Fig. 7a and b), increasing the voucher amount shifted a small proportion of healthcare services utilization from the public to the private sector during the first 1–2 years after the voucher amount increased. In the scenario design, we maintained a similar pace to the current EHCV scheme by implementing an annual voucher amount of $1000 4 years apart. The setting shows a steady increase in the total number of visits to public doctors and the ratio of visits between public and private doctors by year due to population aging and market price inflation from supplier-induced demand. A 0.7 million shift from public-to-private utilization was observed from the first year of increase to $3000, whereas the ratio reduced marginally from 0.72 to 0.64. The impact on the reduction in utilization of public healthcare services was not apparent in later years, wherein the ratio between public and private primary care usage remained approximately 0.80—only a minor difference from that of the baseline scenario.

Compared with the strategy of increasing annual voucher amount, lowering the age eligibility to include those aged 60 years generated a similar effect in reducing reliance on the public sector (Fig. 7a and b). The simulation results showed that the strategy was only able to shift 0.7 million more healthcare visits from the public-to-private sector in the first year of implementation, compared with the baseline scenario. The strategy even tended to be ineffective in later years, presenting results with a marginal 0.05 difference in the public-to-private ratio and only a 0.5 to 1 million shift in visits.

When an additional voucher for chronic conditions follow-up could be offered to elderly people, a significant impact could be observed during the first several years in the post-implementation period (Fig. 7a and b). The enhanced strategy resulted in a shift of 3.2 million visits from public to private primary care, and a reduction in the public-to-private ratio to 0.50, which was more effective than the strategy of increasing the annual voucher amounts in the first year of implementation. However, as with the first strategy of increasing the annual voucher amounts, an additional voucher for chronic conditions follow-up was unsuitable for a long-term planning strategy because the effect on reducing public healthcare utilization was not long-lasting and the public-to-private ratio gradually increased to a higher level, primarily because of population aging and the market price inflation of private healthcare services.

## Discussion

The EHCV scheme is expected to lead to an increase in the number of elderly people selecting private primary healthcare services and reducing reliance on the public sector in Hong Kong, as reported in the literature [20–23, 25]. However, numerous concerns regarding the scheme have been reported—for example, a low uptake rate during the initial implementation phase, an insufficient subsidy amount, and limited utilization of preventive services [20–24]. To this end, improvements in the scheme are required to achieve the intended goal of enhancing a shift in healthcare utilization from public to private. We proposed a framework for SD modeling analysis to examine the long-term effectiveness of numerous potential enhancements of the EHCV scheme. An SD model was formulated to simulate the impact of the different enhanced strategies suggested by the expert panel. The following key research questions are addressed:

### Will the current EHCV scheme, with an annual amount of $2000 for individuals aged 65 years or above, remain sufficient in the long run?

The current EHCV scheme is unable to relieve the burden of over-utilization of public healthcare services as well as the aging trend in the local population. Our simulation results showed that the current scheme was unable to reduce the utilization of the public sector and the ratio of visits between the public and private sectors.

### Should officials increase the voucher amounts in the scheme?

The impact of increasing the voucher amount was not significant for the reduction of reliance on public healthcare utilization. By maintaining a similar pace of amount increment under the current scheme, the strategy could only shift a small proportion of healthcare services utilization from public to private during the first 1 to 2 years after the voucher amount increased, which was likely caused by population aging and market price inflation from supplier-induced demands.

### Should the age eligibility be lowered to include those aged 60 years in the scheme?

Our simulation showed that lowering the age eligibility to 60 years old did not result in a notable reduction in reliance on the public sector. This strategy can only lead to an insignificant difference in the public-to-private ratio in later years.

### Should an additional voucher be introduced for chronic conditions follow-up?

An additional voucher for chronic conditions follow-up can be increasingly effective, compared with the strategy of solely increasing the annual voucher amounts during the first several years in the post-implementation period because of a substantial increase in the utilization of voucher use from the aging population. However, the enhancement effect of reducing public healthcare utilization could not be maintained for a period of time reflected in the steady growth of public-to-private ratio in later years.

The EHCV scheme is a simple means of balancing private-to-public healthcare. Despite multiple increases in voucher amounts in the past, low (as perceived by elderly individuals) subsidy value remained a persistent concern since the implementation of the EHCV. Our simulation investigation results indicate that the current voucher setting was ineffective in reducing reliance on the public sector. However, providing an additional voucher for chronic conditions follow-up to the elderly can result in reduction in the reliance on public healthcare services, particularly for elderly people with chronic conditions, thereby leading to a notably higher proportion of utilizing public healthcare services, compared with those without chronic conditions. Along with the aging trend in the local population, chronic diseases, such as stroke, dementia, and diabetes, as well as cognitive and physical decline, will definitely follow this ongoing demographic change. The effect of the additional voucher for increasing private care usage ensures that capital is invested into the previously lacking chronic disease management of elderly people. This prevents elderly people from paying extra for consultations or expressing unwillingness to pay, particularly for some drugs for chronic conditions, which could be expensive in private clinics or for diseases requiring multiple follow-ups. The effect was not long-lasting in later years, and an enhancement of the voucher amount should thus be considered if long-term planning is required. This strategy requires additional government expenditure, and to this end, improving elderly individuals’ willingness to pay (WTP) was recommended [25]. Improving the perceived benefit of using private healthcare and awareness of the scheme must be promoted as the local elderly population often express concerns regarding the price, quality, and unfamiliarity of private services, thereby significantly limiting their WTP. Liu et al. also showed that elderly individuals were willing to pay more for the same service with an increased subsidy [25].

In this study, we showed that lowering the age eligibility to include those aged 60 years as well as increasing the eligible population in the scheme was ineffective in the long run. One of the reasons was that many elderly people spent their vouchers on general outpatient visits for acute conditions [21–23], whereas younger individuals may not present as many acute conditions as elderly people. Other purposes of utilization were fewer in this group, including preventive services. More health promotion work should be conducted in the local community before considering lowering the age eligibility in the scheme.

The current EHCV scheme in Hong Kong has several concerns; however, these concerns have yet to be addressed. Healthcare vouchers are unlikely to serve as an effective strategy for decreasing public primary care work for increasingly efficient allocation of medical resources. Compared with the U.S. Medicare as a relatively mature health insurance program, the voucher scheme has only been implemented for more than 10 years. In the early phase, when the U.S. government began giving voucher options around the mid-1980s, the participation rate was below 5% [33]. They face many challenges, such as low area coverage, low awareness, and significant variations between different regions and states [11]. The program only began to gain success after years of adjustment, advertising, and government assistance. Although regional variation is not a problem in Hong Kong, the voucher scheme faces similar challenges, such as a lack of service provider participants and voucher expenditure differing from their intended use. Lack of provision in the public sector for long-term planning and “competition” from public healthcare services is also a local problem encountered in running the EHCV scheme. The U.S. Medicare required over 30 years of improvements, and it will certainly take time for Hong Kong to determine the best settings for vouchers to ensure the effectiveness of the scheme. In addition, Medicare is a well-developed health insurance program, whereas EHCV is a scheme that provides financial incentives for elderly people utilizing various private healthcare services. Without quality assurance for the scheme, this undoubtedly led to concerns about exploitation and fraud for the private healthcare industry to generate profit. Criticisms for several issues, such as unnecessary purchases or exorbitant prices for spectacles and eye tests, have been reported [34]. The average claim at optometrists from 2016 to 2019 was between $1600 and $1951, the average claim for general medical practitioners from 2016 to 2019 was between $260 and $330 [31]. Therefore, continuous monitoring and evaluation of the scheme is necessary for reducing abuse by healthcare providers.

Mathematical models evaluating interventions require systematic plausibility, and must thus account for the abstraction and generalization of realism. Therefore, our SD model only captured the necessary variables into the model parameterization that was reviewed in the qualitative phase. Some socio-demographic characteristics (e.g., gender and income level), geographical factors (e.g., accessibility to healthcare facilities and regional heterogeneity), and utilization characteristics (e.g., availability of services and number of healthcare staff) were not included in the model development, in consideration of the balance between model complexity and result generalizability. We acknowledge that some of these factors may affect the simulation results; for example, the lower-income group may have more utilization in the public sector. Although detailed utilization information (e.g., availability of services and number of healthcare staff) as well as case-mix data were unavailable, an additional analysis was conducted to examine the attendance-to-doctor ratio as a measure of workforce pressure to explore the effect of sufficient capacity on our study results by using statistics from a local health manpower survey [35]. According to the results (Additional File 2), when the growth of supply was accounted for, both the ratios of attendance-to-public doctor and attendance-to-private doctor varied marginally; therefore, we speculate that in a plausible change in service capacity, the effects of the tested voucher scenarios remain robust.

Another major limitation is that the unfulfilled needs of the local population were not considered in the model development due to a lack of relevant data [36]. In Hong Kong, limited provision, long waiting times, and limited quotas in public healthcare services may hinder some individuals from visiting public doctors, and they may also be unwilling to visit private doctors due to lower income, particularly for older patients with chronic diseases who require frequent consultation and medication even though they receive healthcare needs. The introduction of the voucher provides an incentive for this group of people to visit private doctors without generating an actual shift from public healthcare services in the healthcare system. Alternatively, we assumed that the provision of public healthcare services could be provided continuously in the long run, thereby leading to the assumption that it may not be conducted efficiently in the future when considering the workforce shortage and shrinking working population in Hong Kong. Considering these reasons, the simulation results could result in an overestimation of the intervention effects.

## Conclusion

Various recommendations have been proposed for improving the current EHCV scheme to reduce the reliance on the public sector in Hong Kong. In this study, we employed an SD modeling approach to examine the effectiveness of some enhanced strategies in the long run. The results showed that it was difficult to relieve the burden of over-utilization of public primary healthcare services along with the aging trend in the local population. Even if the increment rate of the annual amount can be maintained at a similar pace to the historical trends or the age eligibility can be lowered to include those aged 60 years, the enhanced strategies were unable to maintain a steady reduction in the utilization of public healthcare services. Nevertheless, introducing an additional chronic disease-oriented voucher can result in a significant reduction in the reliance on the public sector during the early implementation phase, but the effect cannot be maintained for extended periods because of an increased demand from elderly people requiring more visits for chronic diseases and price inflation from the private market. With a similar idea, the scheme should be redesigned to be more specific or provide a separate voucher that encourages the uptake of under-utilized healthcare services (e.g., preventive care), instead of services designed for unspecified reasons that may also lead to concerns regarding exploitation. Although the original objective of the scheme may not be fully achieved, the voucher can be considered as a subsidy to reduce out-of-pocket payments for elderly people. Therefore, health promotion works encouraging co-payment from elderly people are recommended to avoid increasing government expenditure on substantially improving the voucher scheme.

## Supplementary Information

**Additional file 1.** Variable specifications.

**Additional file 2.** Fig. S1. Ratio of attendance per day to the number of doctors in the public (top) and private (bottom) sectors.

## Data Availability

The datasets generated and/or analyzed in the current study are not publicly available because of confidentiality requirements.

## Abbreviations

DH: Department of Health
EHCV: Elderly healthcare voucher
HKD: Hong Kong dollar
MAPE: Mean absolute percentage error
SD: System dynamics
U.S.: United States
WTP: Willingness to pay
