# Supplementary material for: Long-term effectiveness of elderly health care voucher scheme strategies: a system dynamics simulation analysis
Source: BMC Public Health. 2021 Jun 26;21:1235. doi: 10.1186/s12889-021-11280-z (PMC8236172; doi:10.1186/s12889-021-11280-z)
Supplement: Supplementary file 1 — Additional file 1. Variable specifications. [file 12889_2021_11280_MOESM1_ESM.pdf]

Additional File 1. Variable Specifications

Parameters/ variables	Type*	Values	Definition / Remarks	Sources / Reference
1. Generation of visits to healthcare services				
Population	Level	-	Population size	C&S Department
Birth rate	Fixed	1.1%	Birth rate by year	C&S Department. Estimated by averaging the values from 2009-2017.
Births	Rate	-	Number of new births by year	-
Net movement rate	Fixed	0.2%	Net rate of difference between immigration and emigration	C&S Department. Estimated by averaging the values from 2009-2017.
Net movement	Rate	-	Difference between immigration and emigration	-
Death rate	Fixed	0.6%	Death rate by year	C&S Department. Estimated by averaging the values from 2009-2017.
Deaths	Rate	-	Number of deaths	-
Proportion of individuals aged below 60 years	Fixed	Varied by years	Proportion of individuals aged <60 years by years	C&S Department.
Proportion of individuals aged 60-64 years	Fixed	Varied by years	Proportion of individuals aged 60-64 years by years	C&S Department.
Proportion of individuals aged 65-69 years	Fixed	Varied by years	Proportion of individuals aged 65-69 years by years	C&S Department.
Proportion of individuals aged 70 years or above	Fixed	Varied by years	Proportion of individuals aged >=70 years by years	C&S Department.

Parameters/ variables	Type*	Values	Definition / Remarks	Sources / Reference
Average number of visits per individual aged <60 years	Fixed	0.063	Average number of visits per individual aged <60 years per time	Cross-Sectional Surveys
Average number of visits per individual aged 60-64 years	Fixed	0.118	Average number of visits per individual aged 60-64 years per time	Cross-Sectional Surveys
Average number of visits per individual aged 65-70 years	Fixed	0.147	Average number of visits per individual aged 65-70 years per time	Cross-Sectional Surveys
Average number of visits per individual aged 70 years or above	Fixed	0.342	Average number of visits per individual aged 70 years or above per time	Cross-Sectional Surveys
Total number of visits for individual aged <60 years	Auxiliary	-	Total number of visits for individual aged <60 years per time	-
Total number of visits for individual aged 60-64 years	Auxiliary	-	Total number of visits for individual aged 60-64 years per time	-
Total number of visits for individual aged 65-70 years	Auxiliary	-	Total number of visits for individual aged 65-70 years per time	-
Total number of visits for individual aged 70 years or above	Auxiliary	-	Total number of visits for individual aged 70 years or above per time	-
Baseline proportion of visiting public from individuals aged<60 years	Fixed	44.0%	Baseline proportion of visits to public healthcare services from individuals aged<60 years	Cross-Sectional Surveys
Baseline proportion of visiting public from individuals aged 60-64 years	Fixed	44.0%	Baseline proportion of visits to public healthcare services from individuals aged 60-64 years	Cross-Sectional Surveys

Parameters/ variables	Type*	Values	Definition / Remarks	Sources / Reference
Baseline proportion of visiting public from individuals aged 65-69 years	Fixed	53.7%	Baseline proportion of visits to public healthcare services from individuals aged 65-69 years	Cross-Sectional Surveys
Baseline proportion of visiting public from individuals aged 70 years or above	Fixed	55.0%	Baseline proportion of visits to public healthcare services from individuals aged 70 years or above	Cross-Sectional Surveys
Number of visits to private healthcare services	Auxiliary	-	Total number of visits to private healthcare services	-
Number of visits to public healthcare services	Auxiliary	-	Total number of visits to public healthcare services	-
2. Generation of visits of using EHCV				
Inflation rate from supplier induced demand	Fixed	5% for voucher amount<=1000; 20%, 15%, 10%, and 5% for the first, second, third, and years afterward when voucher amount increased	Annual rate of service price increase from private sector when the voucher amount increases	Administrative data from DH
Voucher amount	Fixed	250 from 2009 to 2011; 500 in 2012; 1000 per year in 2013; 2000 after 2014	Annual voucher amount of the EHCV scheme	-
Adjustment factor for preventive services	Fixed	1 for voucher amount<3000; 1.14 for voucher 3000<=amount<40	Increase of utilization rate for preventive services i.e. chronic condition, dentistry, and vaccination when voucher amount	Cross-Sectional Surveys

Parameters/ variables	Type*	Values	Definition / Remarks	Sources / Reference
		00; 1.27 for voucher 4000<=amount<5000; 1.41 for voucher amount>=5000	increases. Estimated quantile distribution from surveys.	
Proportions of visits for non-preventive services	Auxiliary	-	Proportion of visits for non-preventive services in the eligible population.	Cross-Sectional Surveys
Expected number of voucher visits for non-preventive services	Auxiliary	-	Expected number of voucher visits that can be used for non-preventive services in the eligible population	-
Average amount claimed for non-preventive services	Level	289	Estimated amount (HKD$) claimed for non-preventive services per consultation on average.	Administrative data from DH
Inflation for non-preventive services	Rate	-	Rate of increase for average amount claimed for non-preventive services	-
Proportions of visits for chronic conditions	Fixed	8.6%	Proportion of visits for chronic conditions in the eligible population	Cross-Sectional Surveys
Expected number of voucher visits for chronic conditions	Auxiliary	-	Expected number of voucher visits that can be used for chronic conditions in the eligible population	-
Average amount claimed for chronic conditions	Level	267	Estimated amount (HKD$) claimed for chronic conditions per consultation on average.	Administrative data from DH
Inflation for chronic conditions	Rate	-	Rate of increase for average amount claimed for chronic conditions	-
Proportions of visits for vaccination	Fixed	1.3%	Proportion of visits for vaccination in the eligible population	Cross-Sectional Survey
Expected number of voucher visits for vaccination	Auxiliary	-	Expected number of voucher visits that can be used for vaccination in the eligible population	-

Parameters/ variables	Type*	Values	Definition / Remarks	Sources / Reference
Average amount claimed for vaccination	Level	345	Estimated amount (HKD$) claimed for vaccination per consultation on average	Administrative data from DH
Inflation for vaccination	Rate	-	Rate of increase for average amount claimed for vaccination	-
Proportions of visits for dentistry	Fixed	2.3%	Proportion of visits for dentistry in the eligible population	Cross-Sectional Survey
Expected number of voucher visits for dentistry	Auxiliary	-	Expected number of voucher visits that can be used for dentistry in the eligible population	-
Average amount claimed for dentistry	Level	865	Estimated amount (HKD$) claimed for dentistry per consultation on average	Administrative data from DH
Inflation for dentistry	Rate	-	Rate of increase for average amount claimed for dentistry	-
Proportions of visits for other purposes	Fixed	17.7%	Proportion of visits for other purposes (e.g. allied health) in the eligible population	Cross-Sectional Survey
Expected number of voucher visits for other purposes	Auxiliary	-	Expected number of voucher visits that can be used for other purposes in the eligible population	-
Average amount claimed for other purposes	Level	550	Estimated amount (HKD$) claimed for other purposes per consultation on average	Administrative data from DH
Inflation for other purposes	Rate	-	Rate of increase for average amount claimed for other purposes	-
Expected total number of voucher visits	Auxiliary	-	Total expected number of voucher visits that can be used for all services	-
3. Generation of visits of using vouchers for chronic diseases				
Voucher amount for chronic diseases	Control	Tested with 1,000 and 2,000 per year starting from year 2021	The amount for testing additional voucher for visits to chronic disease conditions. A 3% inflation was assumed by years.	-

**Parameters/ variables**	**Type***	**Values**	**Definition / Remarks**	**Sources / Reference**
Proportion of individuals aged 60-64 years with chronic diseases	Fixed	58%	Proportion of individuals aged 60-64 years with at least one chronic diseases	Thematic Household Survey Report from C&S Department
Proportion of individuals aged 65-69 years with chronic diseases	Fixed	62%	Proportion of individuals aged 65-69 years with at least one chronic diseases	Thematic Household Survey Report from C&S Department
Proportion of individuals aged 70 years or above with chronic diseases	Fixed	70%	Proportion of individuals aged 70 years or above with at least one chronic diseases	Thematic Household Survey Report from C&S Department
Population for voucher of chronic diseases	Auxiliary	-	Total population eligible for voucher of chronic diseases	-
Utilization pattern of voucher of chronic diseases	Fixed	7.25% for voucher amount<=1000; 36.25% for voucher 1000<amount<=2000; 54.38 for voucher 2000<amount<=3600; 72.5% for voucher amount>3600	Expected number of visits for chronic conditions when chronic voucher amount increases. Estimated quantile distribution from surveys. A 3% inflation was assumed by years.	Cross-Sectional Surveys
Voucher visits for chronic conditions	Auxiliary	-	Total number of visits for chronic conditions when voucher of chronic diseases is applied	-
4. Generation of actual number of visits using vouchers				
Eligible population	Auxiliary	-	Population size of all eligible individuals for the scheme	-
EHCV eligible age criteria	Control	70 before July 1, 2017 and 65 after	Eligibility age for the scheme	-

Parameters/ variables	Type*	Values	Definition / Remarks	Sources / Reference
		July 1, 2017; aged >60 years was tested		
Eligible population aged 60-64 years	Auxiliary	-	Population size of individuals aged 60-64 years that eligible for the current scheme	-
Eligible population aged 65-70 years	Auxiliary	-	Population size of individuals aged 65-70 years that eligible for the current scheme	-
Eligible population aged 70 years or above	Auxiliary	-	Population size of individuals aged >=70 years that eligible for the current scheme	-
Utilization rate	Fixed	28% at 2009; 67% at 2013; 84% at 2016 and afterward	Proportion of eligible elderly have ever used the voucher	Cross-Sectional Surveys and DH's published statistics
Actual number of voucher claims	Auxiliary	-	Total number of voucher claims by time	-
Average amount claimed	Fixed	Estimate for calibration	Average amount claimed per visit overall that used for model calibration	-
Expenditure of vouchers	Auxiliary	-	A calibrated total expenditure of vouchers for all services	-
5. Changes in utilization of primary healthcare services				
Change in visits of public healthcare services	Auxiliary	-	Number of visits of public healthcare services when voucher scheme is applied	-
Change in visits of private healthcare services	Auxiliary	-	Number of visits of private healthcare services when voucher scheme is applied	-
Ratio of public visit to private visit	Auxiliary	-	Ratio of elderly visits to public doctors and private doctors which is the primary endpoint of the study.	-

* Fixed: Parameter keeping constant in the model; Auxiliary: Computed variable with a value independent to its previous time; Level: an accumulation variable with a value changing over time; Rate: a flow variable directly change the level variable; Control: control the input values of model simulation
