# Supplementary figures and images for: Long-term effectiveness of elderly health care voucher scheme strategies: a system dynamics simulation analysis
Source: BMC Public Health. 2021 Jun 26;21:1235. doi: 10.1186/s12889-021-11280-z (PMC8236172; doi:10.1186/s12889-021-11280-z)

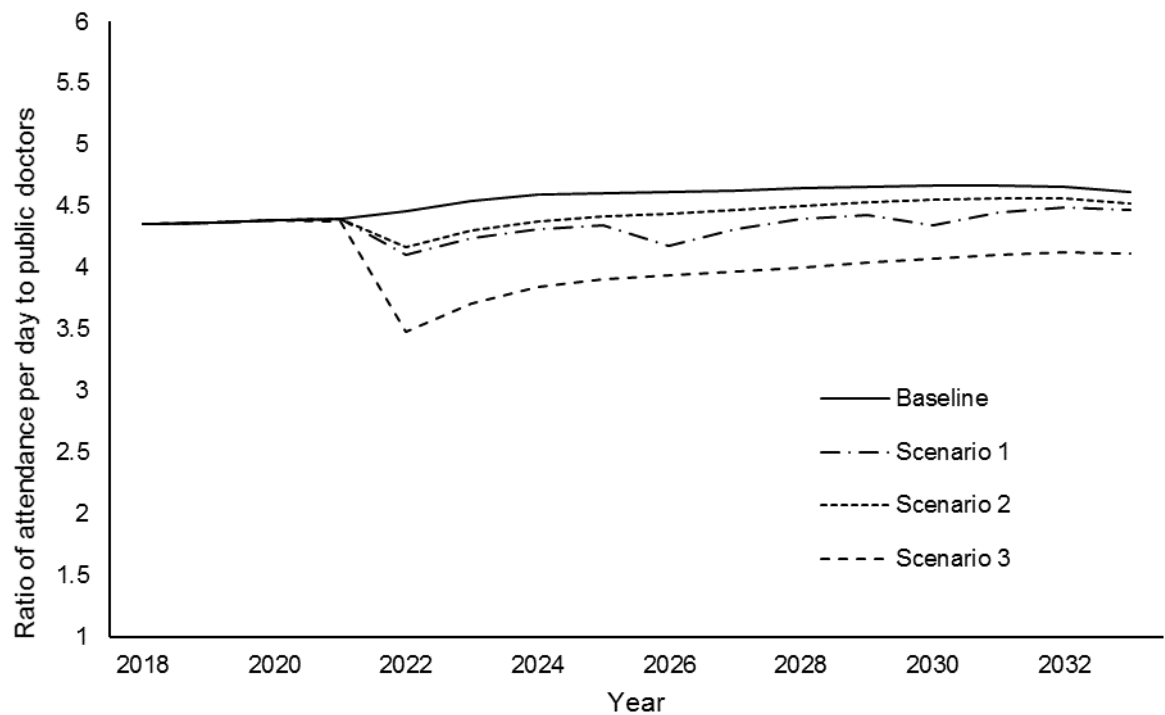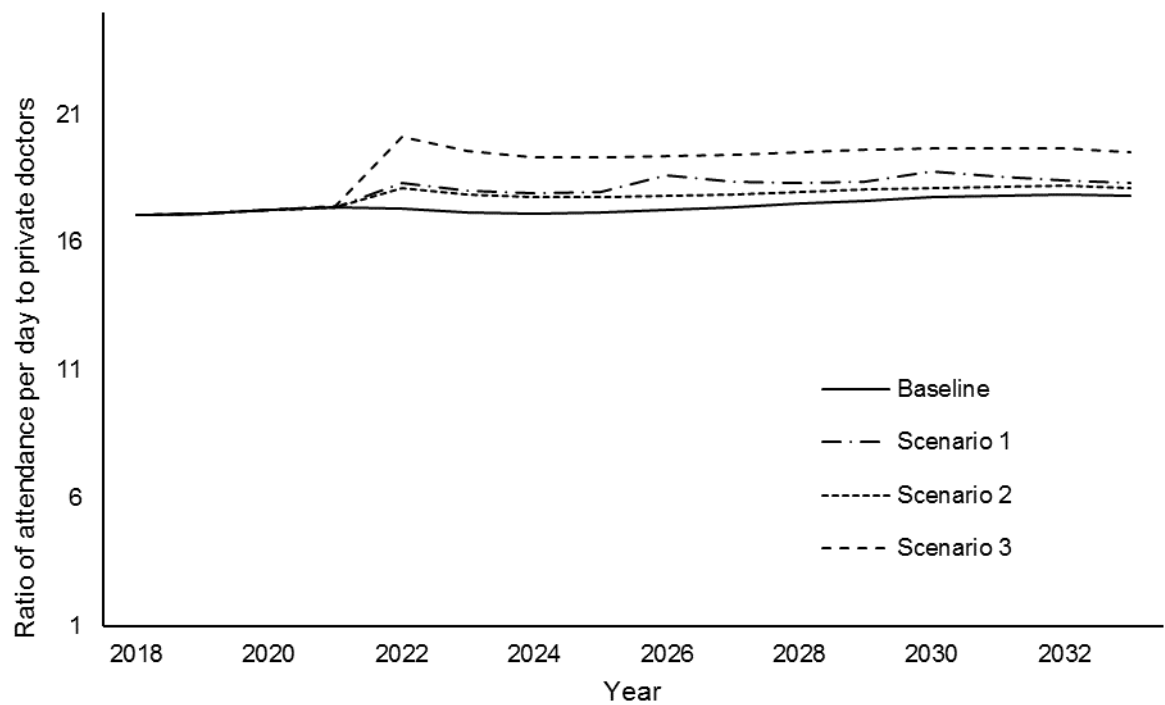

Supplement: Supplementary file 2 — Additional file 2. Fig. S1. Ratio of attendance per day to the number of doctors in the public (top) and private (bottom) sectors. [file 12889_2021_11280_MOESM2_ESM.pdf]
